# Biodiversidata: An Open-Access Biodiversity Database for Uruguay

**DOI:** 10.3897/BDJ.7.e36226

**Published:** 2019-06-20

**Authors:** Florencia Grattarola, Germán Botto, Inés da Rosa, Noelia Gobel, Enrique M. González, Javier González, Daniel Hernández, Gabriel Laufer, Raúl Maneyro, Juan A. Martínez-Lanfranco, Daniel E. Naya, Ana L. Rodales, Lucía Ziegler, Daniel Pincheira-Donoso

**Affiliations:** 1 School of Life Sciences, University of Lincoln, Brayford Campus, Lincoln, United Kingdom School of Life Sciences, University of Lincoln, Brayford Campus Lincoln United Kingdom; 2 Department of Micriobiology and Immunology, Montana State Universitiy, Bozeman, United States of America Department of Micriobiology and Immunology, Montana State Universitiy Bozeman United States of America; 3 Departamento de Métodos Cuantitativos, Facultad de Medicina, Universidad de la República, Montevideo, Uruguay Departamento de Métodos Cuantitativos, Facultad de Medicina, Universidad de la República Montevideo Uruguay; 4 Programa para la Conservación de los Murciélagos de Uruguay, Museo Nacional de Historia Natural, Montevideo, Uruguay Programa para la Conservación de los Murciélagos de Uruguay, Museo Nacional de Historia Natural Montevideo Uruguay; 5 Laboratorio de Sistemática e Historia Natural de Vertebrados, Facultad de Ciencias, Universidad de la República, Montevideo, Uruguay Laboratorio de Sistemática e Historia Natural de Vertebrados, Facultad de Ciencias, Universidad de la República Montevideo Uruguay; 6 Área Biodiversidad y Conservación, Museo Nacional de Historia Natural, Montevideo, Uruguay Área Biodiversidad y Conservación, Museo Nacional de Historia Natural Montevideo Uruguay; 7 Museo Nacional de Historia Natural, Montevideo, Uruguay Museo Nacional de Historia Natural Montevideo Uruguay; 8 NGO JULANA (Jugando en la Naturaleza), Montevideo, Uruguay NGO JULANA (Jugando en la Naturaleza) Montevideo Uruguay; 9 Department of Wildlife, Fisheries and Aquaculture, Mississippi State University, Mississippi, United States of America Department of Wildlife, Fisheries and Aquaculture, Mississippi State University Mississippi United States of America; 10 Departamento de Ecología y Evolución, Facultad de Ciencias, Universidad de la República, Montevideo, Uruguay Departamento de Ecología y Evolución, Facultad de Ciencias, Universidad de la República Montevideo Uruguay; 11 Centro Universitario Regional del Este (CURE), Universidad de la República, Maldonado, Uruguay Centro Universitario Regional del Este (CURE), Universidad de la República Maldonado Uruguay; 12 MacroBiodiversity Lab, School of Science and Technology, Department of Biosciences, Trent University, Nottingham, United Kingdom MacroBiodiversity Lab, School of Science and Technology, Department of Biosciences, Trent University Nottingham United Kingdom

**Keywords:** Uruguay, Biodiversity, Species Occurrence Records, Tetrapods, Amphibia, Reptilia, Aves, Mammalia

## Abstract

**Background:**

The continental and marine territories of Uruguay are characterised by a rich convergence of multiple biogeographic ecoregions of the Neotropics, making this country a peculiar biodiversity spot. However, despite the biological significance of Uruguay for the South American subcontinent, the distribution of biodiversity patterns in this country remain poorly understood, given the severe gaps in available records of geographic species distributions. Currently, national biodiversity datasets are not openly available and, thus, a dominant proportion of the primary biodiversity data produced by researchers and institutions across Uruguay remains highly dispersed and difficult to access for the wider scientific and environmental community. In this paper, we aim to fill this gap by developing the first comprehensive, open-access database of biodiversity records for Uruguay (Biodiversidata), which is the result of a large-scale collaboration involving experts working across the entire range of taxonomic diversity found in the country.

**New information:**

As part of the first phase of Biodiversidata, we here present a comprehensive database of tetrapod occurrence records native from Uruguay, with the latest taxonomic updates. The database provides primary biodiversity data on extant Amphibia, Reptilia, Aves and Mammalia species recorded within the country. The total number of records collated is 69,380, spanning 673 species and it is available at the Zenodo repository: https://doi.org/10.5281/zenodo.2650169. This is the largest and most geographically and taxonomically comprehensive database of Uruguayan tetrapod species available to date and it represents the first open repository for the country.

## Introduction

Uruguay encompasses a peculiar area of South America located within the Pampa Province of the Neotropical Region (Morrone 2014). Both the continental and marine territories currently covered by Uruguay are known to represent rich areas of convergence of diverse environments as heterogeneous as the Amazon, the Pampa, Patagonia and Subantarctic subregions (Morrone 2006, Calliari et al. 2003). For example, Grela (2004) suggests the existence of a phytogeographic longitudinal division of the country’s territory, with a western area characterised by the occurrence of Paranaense and Chaco species and an eastern area marked by different Paranaense species and relicts of flora from the Brazilian Cerrado (Grela and Brussa 2003). Additionally, Arballo and Cravino (1999) and Gonzalez and Martínez-Lanfranco (2010) describe the similarities between the bird and mammal assemblages of Uruguay and the species from adjacent subregions, indicating the spatial convergence of lineages from Brazilian and Andino-Patagonian origins. The reptiles and amphibians, on the other hand, are the result of lineage radiations that come from subregions as contrasting as Patagonia and the Amazon (Pincheira-Donoso 2010). Given these unique biodiversity features, the geographic region, encompassed by the territory of Uruguay, has been proposed to represent a differentiated unit of Pampa, defined by the unique composition of its flora and fauna (Chebataroff 1942, Dos Santos et al. 2016). Therefore, it is surprising that these biogeographic features, combined with the country’s small territorial area (176,220 km2) and its relatively uniform elevational topography (513 m maximum altitude), remain one of the poorest-known across the Americas as a whole. These limitations apply fundamentally to any measure of biodiversity, such as the patterns of distribution of species-richness, endemism and threatened species (Canavero et al. 2010, Soutullo et al. 2013). Collectively, such lack of information hampers any attempts to assess, strategically study and manage the biodiversity and the natural resources of the country.

**Figure 1.**
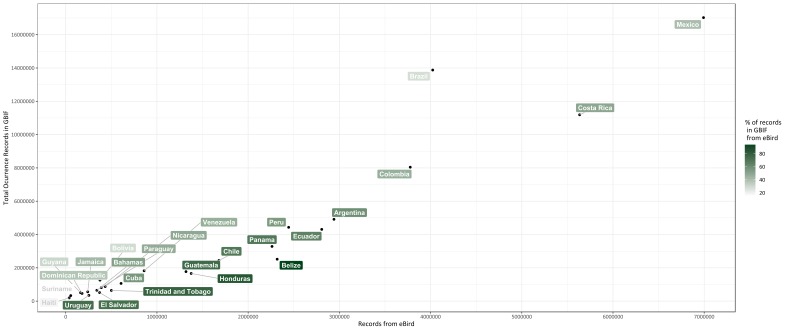
Distribution of the number of occurrence records available in the Global Biodiversity Information Facility (GBIF) (as of 7 June 2019) for each country of Latin America, relative to the number of records that have been submitted by eBird users. The respective proportion is shown in the green scale.

Currently, national biodiversity databases are unavailable and, thus, the dominant proportion of the primary biodiversity data produced in the country is highly dispersed and difficult to access for the wider scientific community and for policy-makers. Likewise, the Global Biodiversity Information Facility (GBIF) reveals that Uruguay ranks amongst the countries of America with the lowest levels of available data on their biodiversity (Fig. 1). In the GBIF platform (as of 7 June 2019), 73.5% of the records belong to the Aves Class, all of which proceed from the eBird initiative. As shown in Fig. 1 , the overwhelming contribution of records provided by eBird to GBIF highlights the enormous role that data, provided by citizens, play in the development of global biodiversity datasets, while at the same time, points out the critical taxonomical biases encountered in GBIF for the region.

### First open biodiversity database of Uruguay

Here, we introduce Biodiversidata, the first database derived from the Uruguayan Consortium of Biodiversity Data (biodiversidata.org), a collaborative initiative aimed at hosting and distributing via an open-access platform a comprehensive database on the biodiversity of Uruguay. The total number of records collated is 69,380, from across 673 species (Table 1). Biodiversidata contains primary biodiversity data (i.e. data records that document the occurrence of a species in space and time) from all the native amphibian, reptile, bird and mammal species recorded in Uruguay to date. Therefore, this paper is the first contribution in a series of phases aimed at improving the knowledge of the biodiversity of Uruguay and, importantly, establishing a fully open-access resource for the wider community from this point on. The data are currently being used to (i) identify spatial patterns of species richness, local endemism and endangerment within tetrapod species of Uruguay, to then assess the spatial congruence amongst these patterns, (ii) quantify the spatial and temporal incompleteness of the inventory and (iii) identify high priority areas of historically poor sampling (‘hotspots of ignorance’), with the ultimate aim of facilitating the development of future sampling strategies and efforts to complete these gaps. This database, therefore, has been generated, based on the principle that collaboration amongst experts can strongly push forward the development of fields and, in this particular case, improve our knowledge on the biodiversity of Uruguay by overcoming data-scarcity and enriching the understanding of regional and larger-scale biodiversity patterns. Collectively, Biodiversidata offers the first open biodiversity repository for the country and the most comprehensive geographically and taxonomically resource for biodiversity and environmental studies in Uruguay to date.

## Sampling methods

### Sampling description

The database was developed, based on the collection of data from a range of different sources. A significant proportion of the data was collected by expert members of Biodiversidata. These records can be found with the value ‘Unpublished data’ under the term ‘associatedReference’. A proportion of them has been deposited in national specimen collections such as the Mammalogy collection of the Museo Nacional de Historia Natural of Uruguay and the Vertebrate collection of the Facultad de Ciencias, Universidad de la República (Uruguay). In addition to the large volume of original data, we have also incorporated all readily available records from multiple sources, including online databases (i.e. GBIF) as well as data currently published but not available in the format of other sources of compiled information. These include data from primarily field guides and books and primary literature such as monographs, systematic accounts, species descriptions, reviews and reports of range extensions, in journals such as “Check List” and the local “Boletín de la Sociedad Zoológica del Uruguay”, amongst others. A complete list of sources for the occurrence records is shown in Table 2. Most of the sources used are freely available online, while numerous other literature resources that document the primary biodiversity data of Uruguay still remain inaccessible.

The GBIF dataset was obtained by searching for Uruguay in the ‘country or area’ field (as for 15 January 2018), retrieving 185,519 occurrences from 573 datasets, including 8,925 species of Animalia, Plantae, Fungi, Bacteria, Chromista, Protozoa and Archaea. These data on species occurrences are available on the GBIF portal at https://doi.org/10.15468/dl.dmul8x. Most of these records were submitted by the eBird project (56.1% of the total amount) and the rest derive from diverse specimen collections around the world, such as the National Museum of Natural History Smithsonian Institution (4.2%), the Instituto de Botánica Darwinion in Argentina (3.9%), the Missouri Botanical Garden’s Herbarium (3%), the Museo Argentino de Ciencias Naturales "Bernardino Rivadavia" (2.1%), the Swedish Museum of Natural History (1.6%), the Museum of Comparative Zoology at Harvard University (0.72%), the American Museum of Natural History of New York (0.68%) and the Natural History Museum of London (0.57%). None of the records was submitted by Uruguayan institutions, most likely because of the major public sources of specimen biodiversity information (government and academia) are not open nor publicly available.

### Quality control

Different methods were applied to treat the data derived from each of the above-mentioned sources. For the GBIF data, only records of amphibians, reptiles, birds and mammals were included in this first version. Exotic species and records without complete date of collection/observation or geographic location information were excluded. The data from literature were manually extracted and added to the data collected by members of Biodiversidata. These records were controlled by collection and catalogue number to check their complete independence from the GBIF data. To avoid pseudo-replication in posterior analyses, records were filtered by considering only one record per locality/year. If more than one organism of the same species was collected in a locality in the same year (i.e. same geographic coordinates), we kept the first and most complete record (i.e. the most informative record for the year).

In line with FAIR data Principles (Wilkinson et al. 2016), the database was prepared to improve the findability, accessibility, interoperability and reuse of the data collated. We manually adapted the data following the Darwin Core Biodiversity Data Standard (DwC) (Wieczorek et al. 2012), incorporating 32 descriptive terms (see Data resources section for a full description of each column heading). Likewise, we created a persistent and global identifier for each record, included well-described metadata and applied the most accessible usage licence to the data.

A significant number of the data lacked crucial information in terms of taxon, time and place of collection/observation, a common issue with observational and specimen data (Peterson et al. 2018). Thus, the treating of the vastly heterogeneous records included updating scientific names inconsistencies and the georeferencing of sampling locations when sufficient information was provided. For standardisation of species names and complete taxonomic categories retrieval, we used the R package 'taxize' (Chamberlain et al. 2018). We followed the Integrated Taxonomic Information System database (itis.gov) and the specific reference according to the taxonomic group: Amphibian Species of the World of the American Museum of Natural History, BirdLife International, The Mammal Species of The World and The Reptile Database. For conservation status retrieval according to the IUCN Red List, we used the R package 'rredlist' (Chamberlain 2018). The R scripts used can be found at Grattarola (2019). Georeferenced point data resulted from either GPS measurements, direct estimates of the latitude and longitude of an observation when route and kilometre number data were available or by defining the latitude and longitude of the event locality through the GeoNames Gazetteer database (geonames.org). The details of how geographic latitude and longitude were obtained can be found under the term ‘georeferenceSources’.

## Geographic coverage

### Description

**Figure 2.**
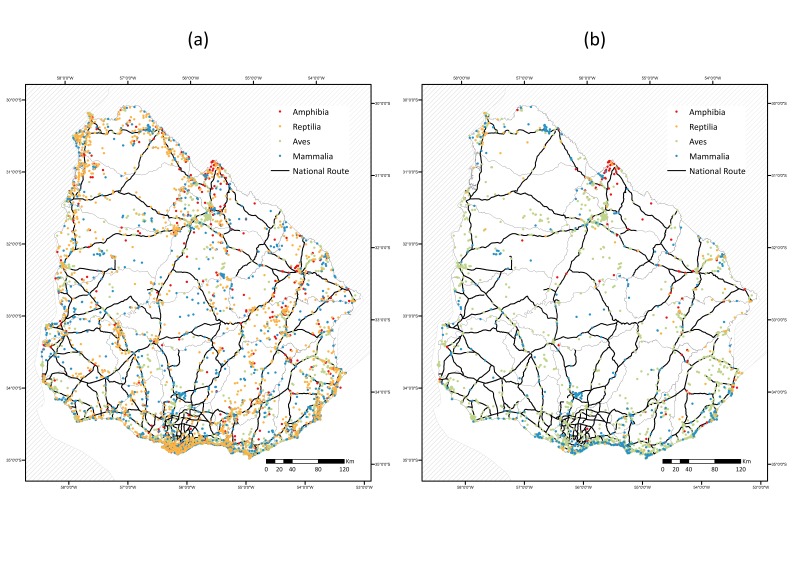
Distribution of the (a) total number of occurrence records (N = 69,380) from species of amphibians, reptiles, birds and mammals from Uruguay and (b) occurrence records from the last 30 years. National routes are shown in black. Projection WGS1984 UTM zone 21S.

The database includes all native and extant species of tetrapods reported in any area within the borders of Uruguay. The occurrence records are not evenly distributed through space as a result of oversampling in some areas and of limited (or no) sampling in other areas (Fig. 2a). When we consider the records of the last 30 years, the geographic coverage amongst groups reduces enormously and becomes dominated by birds (Fig. 2b).

Higher numbers of records are seen in the coast area, whilst the centre of the country holds low sampling densities. The most sampled area of Uruguay is in Montevideo (the capital of the country), followed by the surroundings of Maldonado and Rocha cities, all Atlantic coast areas. We observed this pattern particularly in Aves which, despite being the most sampled group, with 87.4% of the database records, they are strongly spatially biased. Reptiles, on the other hand, with the least number of records in the database, cover the Uruguayan territory better than any other tetrapod group.

After our data collation, we can observe some areas of the country that remain systematically ignored. This disparity in sampling is mostly due to the lack of systematisation in the efforts of zoological exploration of the national territory and responds to the realisation of research projects, faunistic inventories or intensive occasional sampling in a few locations, generally near the main population centres or close to easily accessible areas (Carreira et al. 2005, Soutullo et al. 2013). As can be seen in Fig. 2, areas with more sampling effort tend to be located adjacent to national routes. Nevertheless, this is the first country-wide effort aimed at tackling biodiversity data being lost. In the future, there is substantial work to be done on digitisation and tactical direction of new sampling efforts to enhance the territorial coverage to develop a more accurate picture of the distribution of biodiversity in the country. Therefore, a critical first contribution of the Uruguayan Consortium of Biodiversity Data will involve establishing areas where efforts are urgently needed at the expense of areas that have been historically oversampled.

### Coordinates

-34.973188 and -30.10818 Latitude; -58.43882 and -53.266525 Longitude.

## Taxonomic coverage

### Description

**Figure 3.**
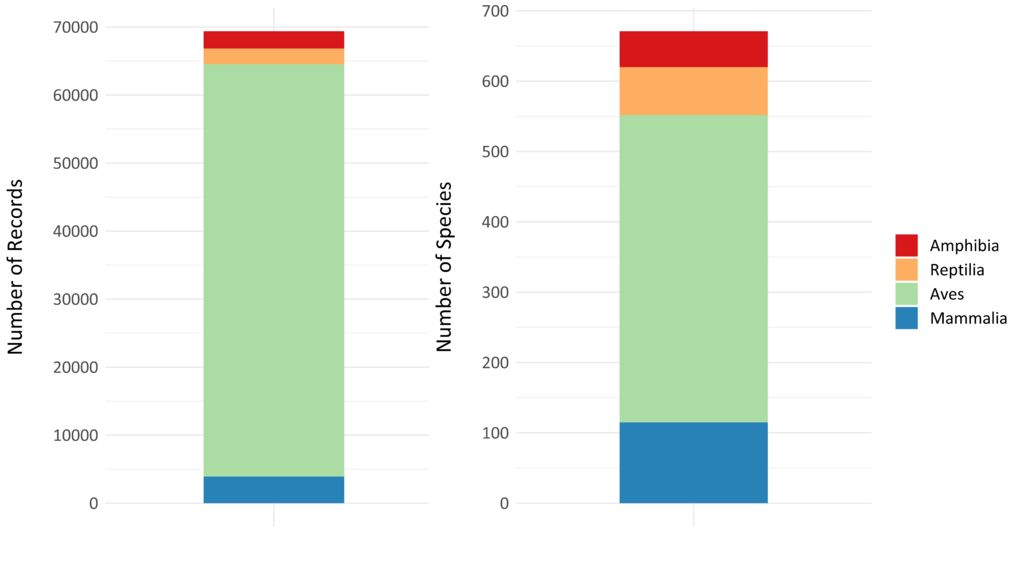
Distribution of the number of occurrence records and number of species collated for each class of the tetrapod group.

The database incudes 69,380, representing 129 families, 446 genera and 673 species: 51 amphibians, 68 reptiles, 437 birds and 117 mammals. The taxonomic coverage is uneven (Fig. 3). For instance, ten bird species make up to 14% of the database records, while 10% of the tetrapod species have only been observed/collected once. Likewise, occurrence records within groups are dominated by their most sampled species, such as *Boana pulchella* (N = 248) and *Pseudis minuta* (N = 195) in Amphibia, *Philodryas patagoniensis* (N = 176) and *Erythrolamprus poecilogyrus* (N = 139) in Reptilia, *Pitangus sulphuratus* (N = 1191) and *Furnarius rufus* (N = 1180) in Aves and *Akodon azarae* (N = 207) and Scapteromys tumidus (N = 187) in Mammalia.

### Taxa included

**Table:** 

Rank	Scientific Name	Common Name
kingdom	Animalia	Animals
subkingdom	Eumetazoa	
phylum	Chordata	
subphylum	Vertebrata	
superclass	Tetrapoda	
class	Amphibia	Amphibians
class	Reptilia	Reptiles
class	Aves	Birds
class	Mammalia	Mammals

## Temporal coverage

### Notes

**Figure 4.**
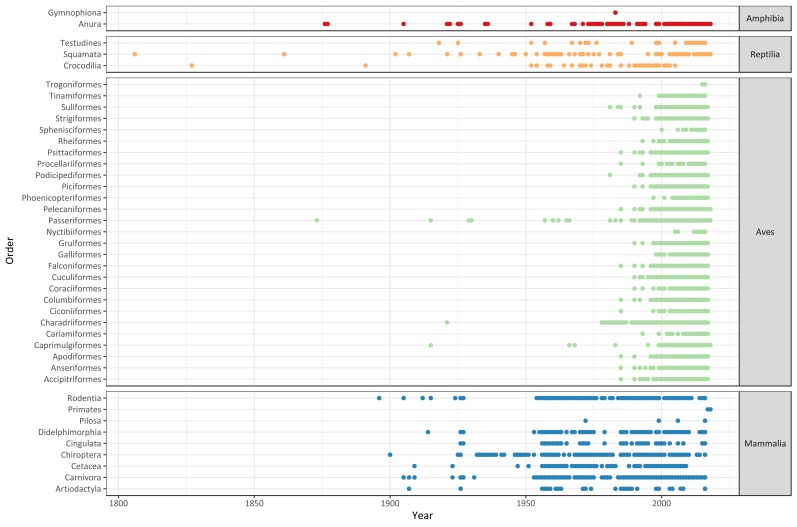
Occurrence records of tetrapod orders reported in Uruguay over time, divided by amphibians, reptiles, birds and mammals.

The records included in Biodiversidata cover samples reported in Uruguay during the period of 1806–2018 (Fig. 4). We observed that occurrence records have been collected mostly intermittently within groups, with a continuously increasing tendency since the beginning of the 20th Century. The steady increase towards the latter half of the century is in part a result of the creation of the School of Science (1945) and several field work expeditions during the next decades that resulted in an increase in the production of research articles (Soutullo et al. 2013). In the case of the records collected from literature, there was a high number lacking date of collection or observation. For instance, a large number of the records collated from Carreira et al. (2005), a detailed scientific monograph on the reptiles of Uruguay, provides location but no date associated to the records. We aim to promote the need to associate spatial records to dates of collection of the datapoints, as this approach is expected to facilitate the development of scientific-based decisions when implementing environmental policies (Peterson et al. 2018). Overall, as was mentioned above, numerous other literature sources and specimens recorded in the country yet need to become digitally accessible, hence, Uruguay will face a great challenge in "rescuing" these data in the future to prevent them being lost.

In particular, bird occurrence records are disproportionally superior in the database (i.e. 87.4% of total number of records), presenting an intense period of sampling effort between 2000 and 2016, mostly derived from citizen science efforts from eBird users (collected from GBIF). Regardless of the spatial bias of these records, it is valuable to note the significant contribution of local ornithologists and birdwatchers (i.e. Aves Uruguay) to the international initiative, which probably stands as the richest and oldest practice of data-sharing known in Uruguay.

## Usage rights

### Use license

Creative Commons Public Domain Waiver (CC-Zero)

## Data resources

### Data package title

Biodiversidata

### Number of data sets

1

### Data set 1.

#### Data set name

Biodiversidata: An Open-Access Database for the Biodiversity of Uruguay: 1806-2018

#### Data format

Darwin Core Archive

#### Number of columns

32

#### Character set

UTF-8

#### Download URL

doi.org/10.5281/zenodo.2650169

#### Data format version

1.0

#### Description

The dataset provides primary biodiversity data on extant Amphibia, Reptilia, Aves and Mammalia species recorded within the country area between 1806-2018. The total number of records collated is 69,380, including 673 species. Suppl. material 1

**Data set 1.** 

Column label	Column description
occurrenceID	An identifier for the Occurrence (as opposed to a particular digital record of the occurrence), constructed from a combination of identifiers in the record that will most closely make the occurrenceID globally unique.
scientificName	The full scientific name, with authorship and date information
scientificNameAuthorship	The authorship information for the scientificName
vernacularName	Common or vernacular name in Uruguay (in Spanish)
kingdom	The full scientific name of the kingdom in which the taxon is classified
phylum	The full scientific name of the phylum or division in which the taxon is classified
class	The full scientific name of the class in which the taxon is classified
order	The full scientific name of the order in which the taxon is classified
family	The full scientific name of the family in which the taxon is classified
genus	The full scientific name of the genus in which the taxon is classified
specificEpithet	The name of the first or species epithet of the scientificName
infraspecificEpithet	The name of the lowest or terminal infraspecific epithet of the scientificName, excluding any rank designation
countryCode	The standard code for the country in which the Location occurs
stateProvince	The name of the next smaller administrative region than country (department) in which the Location occurs
verbatimLocality	The original textual description of the place
decimalLatitude	The geographic latitude (in decimal degrees)
decimalLongitude	The geographic longitude (in decimal degrees)
georeferenceSources	A list of maps, gazetteers or other resources used to georeference the Location
georeferencedBy	A person, group or organisation who determined the georeference (spatial representation) for the Location.
eventDate	The date when the event was recorded. Format: dd-mm-yyyy
year	The four-digit year in which the Event occurred. Format: yyyy
month	The ordinal month in which the Event occurred. Format: mm
day	The integer day of the month on which the Event occurred. Format: dd
basisOfRecord	The specific nature of the data record
institutionCode	The name (or acronym) in use by the institution having custody of the object(s) or information referred to in the record
collectionCode	The name or acronym identifying the collection or dataset from which the record was derived
catalogNumber	An identifier (preferably unique) for the record within the dataset or collection
recordedBy	A list (concatenated and separated) of names of people, groups or organisations responsible for recording the original Occurrence
recordNumber	An identifier given to the Occurrence at the time it was recorded. Often serves as a link between field notes and an Occurrence record, such as a specimen collector's number.
identifiedBy	A list (concatenated and separated) of names of people, groups or organisations who assigned the Taxon to the subject
dynamicProperties	Structured content about the record key:value encoding IUCN red list category of the taxon at the Global level
associatedReferences	A list (concatenated and separated) of identifiers (publication, bibliographic reference, global unique identifier, URI) of literature associated with the Occurrence

## Additional information

The Uruguayan Consortium of Biodiversity Data, is a collaborative association of experts whose aim is to improve Uruguay’s biodiversity knowledge. It was created in 2018 by Florencia Grattarola as part of her PhD project. Its open-access platform (biodiversidata.org) aims to make available the biodiversity data of Uruguay by integrating a broad range of resources including databases, publications, maps, reports and infographics, derived from the work of the team members. The database presented in this study and the original research that is currently emerging from it are the first products of the initiative and will be available in the platform. The database may continue to be updated with new records periodically; check the Zenodo repository for the latest version: https://doi.org/10.5281/zenodo.2650169.

## Supplementary Material

Supplementary material 1 Data for tetrapods occurrence records of Uruguay Data type: Primary biodiversity data
Brief description: Tab-delimited csv data file and xml metadata file corresponding to the 69,380 species occurrence records held in the database
File: oo_311640.zip Florencia Grattarola

## Figures and Tables

**Table 1.** Records collected per tetrapod class showing: number of occurrence records (non-duplicated records/location/year), total number of species, records without information of the date of collection and records collected in the last 30 years, with percentage in parentheses.

	**Number of Occurrence Records**	**Number of Species**	**Records without Date (%)**	**Records from the last 30 years (%)**
Amphibia	2,530	51	1,780 (70.4)	683 (27.0)
Reptilia	2,308	68	1,999 (86.6)	224 (9.7)
Aves	60,627	437	131 (0.2)	60,308 (99.5)
Mammalia	3,915	117	1,687 (43.1)	1,122 (28.7)
Total	69,380	673		

**Table 2.** List of sources used to build the dataset, including the source type and the number of records extracted from each of the sources.

**Source**	**Source type**	**Number of records**	**Groups**
Abreu (2015)	Journal Article	8	Aves
Andrade-Núñez and Aide (2010)	Journal Article	13	Mammalia
Azpiroz et al. (2012)	Journal Article	54	Aves
Bardier and Maneyro (2015)	Journal Article	20	Amphibia
Borteiro et al. (2006)	Journal Article	70	Reptilia
Borteiro et al. (2009)	Journal Article	1	Reptilia
Borteiro et al. (2013)	Journal Article	4	Reptilia
Borteiro et al. (2015)	Journal Article	13	Reptilia
Bou (2013)	Thesis	86	Mammalia
Carreira and Achaval (2007)	Journal Article	2	Reptilia
Carreira and Lombardo (2006)	Journal Article	1	Reptilia
Carreira et al. (2005)	Book	1880	Reptilia
Carreira et al. (2012)	Journal Article	2	Reptilia
Colina et al. (2012)	Journal Article	1	Reptilia
da Rosa	This study	13	Amphibia + Reptilia
de Giorgi Peirano (2016)	Thesis	67	Aves
Elgue and Maneyro (2017)	Journal Article	1	Amphibia
GBIF.org	Online Database	58355	Amphibia + Reptilia + Aves + Mammalia
Gobel & Laufer	This study	285	Amphibia + Reptilia
González-Paredes et al. (2017)	Journal Article	3	Reptilia
González & González	This study	1848	Mammalia
Grattarola	This study	53	Mammalia
Grattarola (2015)	Thesis	36	Mammalia
Hernández	This study	944	Aves
Kolenc et al. (2009)	Journal Article	2	Amphibia
Kolenc et al. (2012)	Journal Article	2	Amphibia
Kwet et al. (2002)	Journal Article	1	Amphibia
Lareschi et al. (2006)	Journal Article	11	Mammalia
Laufer et al. (2009)	Journal Article	2	Amphibia
Maneyro et al. (2008)	Journal Article	2	Amphibia
Maneyro	This study	165	Amphibia + Reptilia
Martínez-Lanfranco et al. (2010)	Journal Article	6	Mammalia
Martínez-Lanfranco	This study	1712	Aves + Mammalia
Masciadri et al. (2007)	Conference Paper	48	Amphibia + Reptilia + Aves
Montero (2016)	Journal Article	85	Reptilia
Naya and Achaval (2006)	Journal Article	5	Mammalia
Naya	This study	220	Aves
Núñez et al. (2004)	Book	1764	Amphibia
Prigioni et al. (2011)	Journal Article	43	Amphibia + Reptilia
Prigioni et al. (2013)	Journal Article	18	Reptilia
Prigioni et al. (2018)	Journal Article	3	Mammalia
Queirolo (2016)	Journal Article	1041	Mammalia
Rodales, Botto & González	This study	91	Mammalia
Rodríguez-Cajarville et al. (2017)	Journal Article	4	Aves
Rodríguez-Mazzini et al. 2001	Report	151	Amphibia + Reptilia + Mammalia
Santana et al. (2013)	Journal Article	1	Amphibia
Sarroca et al. 2009	Report	189	Amphibia + Reptilia + Aves + Mammalia
Vaz-Canosa and Rodríguez-Cajarville (2015)	Journal Article	1	Aves
Velasco-Charpentier et al. (2016)	Journal Article	4	Reptilia
Verrastro et al. (2006)	Journal Article	3	Reptilia
Verrastro et al. (2017)	Journal Article	1	Reptilia
Villamil (2014)	Journal Article	15	Reptilia
Ziegler	This study	31	Amphibia
